# Influence of extracorporeal membrane oxygenation on the pharmacokinetics of ceftolozane/tazobactam: an ex vivo and in vivo study

**DOI:** 10.1186/s12967-020-02381-1

**Published:** 2020-05-27

**Authors:** Camille Mané, Clément Delmas, Jean Porterie, Géraldine Jourdan, Patrick Verwaerde, Bertrand Marcheix, Didier Concordet, Bernard Georges, Stéphanie Ruiz, Peggy Gandia

**Affiliations:** 1 Pharmacokinetics and Toxicology Laboratory, Toulouse University Hospital, Toulouse, France; 2 INTHERES, INRAE, ENVT, Université de Toulouse, Toulouse, France; 3 Intensive Cardiac Care Unit, Rangueil University Hospital, Toulouse, France; 4 Institute of Metabolic and Cardiovascular Diseases (I2MC), UMR-1048, National Institute of Health and Medical Research (INSERM), Toulouse, France; 5 Cardiovascular Surgery Unit, Rangueil University Hospital, Toulouse, France; 6 Critical and Intensive Care Unit, Stomalab UMR 5273 CNRS/UPS-EFS-ENVT-INSERM U1031, Toulouse School of Veterinary Medicine, Toulouse, France; 7 Anesthesia-Emergency-Intensive Care Department, UPEC/IMRB-Inserm U955, Alfort School of Veterinary Medicine, Maisons-Alfort, France; 8 Anesthesia-General Intensive Care Division, Rangueil General Intensive Care Department, Toulouse University Hospital, Toulouse, France

**Keywords:** Ceftolozane, Tazobactam, Beta-lactam, Pharmacokinetics, ECMO

## Abstract

**Background:**

Extracorporeal membrane oxygenation (ECMO) is increasingly used in intensive care units and can modify drug pharmacokinetics and lead to under-exposure associated with treatment failure. Ceftolozane/tazobactam is an antibiotic combination used for complicated infections in critically ill patients. Launched in 2015, sparse data are available on the influence of ECMO on the pharmacokinetics of ceftolozane/tazobactam. The aim of the present study was to determine the influence of ECMO on the pharmacokinetics of ceftolozane-tazobactam.

**Methods:**

An ex vivo model (closed-loop ECMO circuits primed with human whole blood) was used to study adsorption during 8-h inter-dose intervals over a 24-h period (for all three ceftolozane/tazobactam injections) with eight samples per inter-dose interval. Two different dosages of ceftolozane/tazobactam injection were studied and a control (whole blood spiked with ceftolozane/tazobactam in a glass tube) was performed. An in vivo porcine model was developed with a 1-h infusion of ceftolozane–tazobactam and concentration monitoring for 11 h. Pigs undergoing ECMO were compared with a control group. Pharmacokinetic analysis of in vivo data (non-compartmental analysis and non-linear mixed effects modelling) was performed to determine the influence of ECMO.

**Results:**

With the ex vivo model, variations in concentration ranged from − 5.73 to 1.26% and from − 12.95 to − 2.89% respectively for ceftolozane (concentrations ranging from 20 to 180 mg/l) and tazobactam (concentrations ranging from 10 to 75 mg/l) after 8 h. In vivo pharmacokinetic exploration showed that ECMO induces a significant decrease of 37% for tazobactam clearance without significant modification in the pharmacokinetics of ceftolozane, probably due to a small cohort size.

**Conclusions:**

Considering that the influence of ECMO on the pharmacokinetics of ceftolozane/tazobactam is not clinically significant, normal ceftolozane and tazobactam dosing in critically ill patients should be effective for patients undergoing ECMO.

## Background

Extracorporeal membrane oxygenation (ECMO) is a temporary life support technique used to aid respiratory and/or cardiac function in case of organ failure such as acute respiratory distress syndrome or refractory cardiogenic shock [1]. The use of ECMO has considerably increased in adult intensive care units (ICU) due to the improvement in the risk–benefit profile as a result of advances in extracorporeal technology [1, 2]. The putative influence of ECMO on drug pharmacokinetics (PK) is based on three major mechanisms: drug extraction by adsorption on ECMO components, an increase in distribution volume and altered drug clearance [3–5].

Ceftolozane/tazobactam (C/T) (Zerbaxa, Merck & Co., Kenilworth, USA), a novel cephalosporin/beta-lactamase inhibitor combination, is effective against multi-drug resistant strains of *Pseudomonas aeruginosa* and many extended spectrum beta-lactamase (ESBL)-producing Gram-negative bacilli [6]. The C/T combination is a last-line antibiotic treatment for which exposure must be sufficient to ensure microbiological/clinical efficacy. However, the C/T combination was launched in 2015. Therefore, few general data are available and more precisely, data concerning the influence of ECMO on the pharmacokinetics of C/T. A study conducted by Cies et al. [7] reported a major loss of ceftolozane (40 to 60% in 5 h and ~ 90% in 24 h) using an ex vivo model. This information is particularly concerning for clinicians as it suggests a decrease in drug exposure that is enough to lead to therapeutic failure in the absence of any dose adjustment. However, these results provide only partial information on the influence of ECMO since adsorption is not the only modification reported in real life. In fact, inconsistent results of ex vivo and clinical studies on meropenem have been reported [4, 8–10]. Contrary to Cies’ results, the kinetic profile of a patient undergoing ECMO and treated with C/T after lung transplant was similar to the PK profiles observed in patients without ECMO, suggesting no C/T loss [7, 11].

The aim of our study was to determine the influence of ECMO on C/T PK based on two successive and complementary studies: (i) an ex vivo study to document the mechanism of expected ceftolozane adsorption and (ii) an in vivo study for a more general exploration of the consequences of ECMO on the pharmacokinetics of C/T.

## Methods

### Experiments on the ex vivo model

Adult ECMO circuits including 3/8-in. polyvinylchloride tubing, a Revolution centrifugal pump and an EOS ECMO oxygenator (Sorin Group, LivaNova, London, United Kingdom) were maintained in a closed loop. Circuits were primed with normal saline solution which was then exchanged with fresh human blood (24 h old), provided by the Établissement Français du Sang (Toulouse, France). The temperature was set at 37 °C, the pH was maintained between 7.20 and 7.50, and the flow rate between 2.5 and 3 l/min and unfractionated heparin was added to mimic conditions observed in patients undergoing ECMO. Pre-oxygenator C/T boluses (ZERBAXA^®^, MSD, France) were injected at T0, T8 and T16. Post-oxygenator blood samples were collected over the 8-h inter-dose interval each time (T0, T0.5, T1, T2, T3, T4, T6 and T8 post administration). A total of 24 samples were collected.

The same protocol was repeated three times *per* group for two groups: low (n = 3) and high concentrations (n = 3) corresponding to a 9 mg/4.5 mg and 25 mg/12.5 mg C/T dose.

Controls were prepared with glass tubes containing whole blood spiked with C/T in order to achieve the same C/T concentrations aimed at in the ECMO circuits [low (n = 3) and high concentrations (n = 3)]. For each control, nine blood samples were collected (T0, T0.5, T1, T2, T3, T4, T6, T8) in order to determine spontaneous C/T degradation.

C/T concentrations were quantified by a validated liquid chromatography–tandem mass spectrometry method using a Kinetex-Polar-C18 column (Phenomenex, Le Pecq, France) on a Prominence HPLC System (Shimadzu, Marne-la-Vallée, France) coupled with a QTRAP^®^ 4500 (SCIEX, Villebon-sur-Yvette, France). For both molecules, the method was accurate and precise at a linearity range of 0.4–200 mg/l and 0.1–200 mg/l for ceftolozane and tazobactam, respectively. Intra-day and inter-day assay variability were below 10% for all control samples.

For each group studied (i.e. low and high concentration), differences in drug concentrations and recovery over the period studied were assessed for each inter-dose interval (T0–T8, T8–T16 and T16–T24) and the associated controls. To calculate the percentage of drug loss during the inter-dose interval, the difference between the concentration at the beginning and at the end of the inter-dose interval was divided by the concentration at the beginning of the inter-dose interval.

### Experiments on the in vivo porcine model

All experiments were conducted with the approval of the Ethics Committee in the field of animal studies and handling of the animals was according to European guidelines. Six anesthetized and ventilated pigs (Landrace × White Large, 70 kg) were divided into an ECMO group (n = 3) and a control group (n = 3). Anesthesia was induced by ketamine/azaperone then maintained by propofol/midazolam/sufentanyl/cisatracurium in continuous infusion. Doses were adjusted according to the clinical response.

Femoral venoarterial ECMO was implanted. Anticoagulation was ensured by unfractionated heparin; the flow rate was maintained between 3 and 4 l/min, and the gas flow was adapted between 1.5 and 2.5 l/min.

C/T (2 g/1 g) was administered in a 1-h infusion. Sixteen blood samples were collected *per* animal: T0 (before the initiation of the infusion), T0.25, T0.5, T0.75, T1, T1.5, T2 and then every hour from T3 to T11. The same analytical method was used to determine C/T concentrations.

### Pharmacokinetic analysis

Pharmacokinetic exploration was performed by two conventional approaches, i.e. non-compartmental analysis using PK Solver software [12] and compartmental modeling using MONOLIX software 2018 R2 (Lixoft, Antony, France). Different approaches were tested, including one- or two-compartment modeling, to describe the C/T kinetic profile, while proportional and combined (additive + proportional) modeling were tested to describe the residual variability. Once the null model (i.e. model without factors explaining inter-individual variability) was selected, two documented covariates (ECMO and sex) were tested.

Model evaluation was based on the usual criteria: improvement of the likelihood, precision of the PK parameter estimation (relative standard error), diagnostic plot evaluation (observed vs. predicted concentrations; residual plots) and visual predictive check. The significant influence of a covariate to explain inter-individual variability was determined applying the likelihood ratio test (LRT).

The modeling methodology is more precisely described in Additional file 1.

### Statistical analysis

Data are presented as means with standard deviations (SDs) for continuous variables.

Differences in drug concentrations and recovery over the period studied in the ex vivo model were assessed for each inter-dose interval (T0–T8, T8–T16 and T16–T24) and the associated control using a two-sided Dunnett test. A p < 0.05 was considered statistically significant. These statistical analyses were performed using Prism v. 6 (GraphPad Software, San Diego, USA).

To test potential covariate significance, likelihood ratio tests (LRT) were performed. A decrease of more than 3.84 in the likelihood ratio (p-value = 0.05, $\chi^{2}$ distribution, 1 degree of freedom) was considered significant. These statistical analyses were performed using MONOLIX software 2018 R2 (Lixoft, Antony, France).

## Results

### Experiments on the ex vivo model

In all, 144 samples were collected from the six ECMO circuits and 54 samples from the six controls tubes. Three samples from the circuits had incoherent concentrations due to insufficient homogenization time between injection and sampling. Therefore, a total of 141 ceftolozane and tazobactam concentrations from the circuits were exploited.

**Fig. 1:**
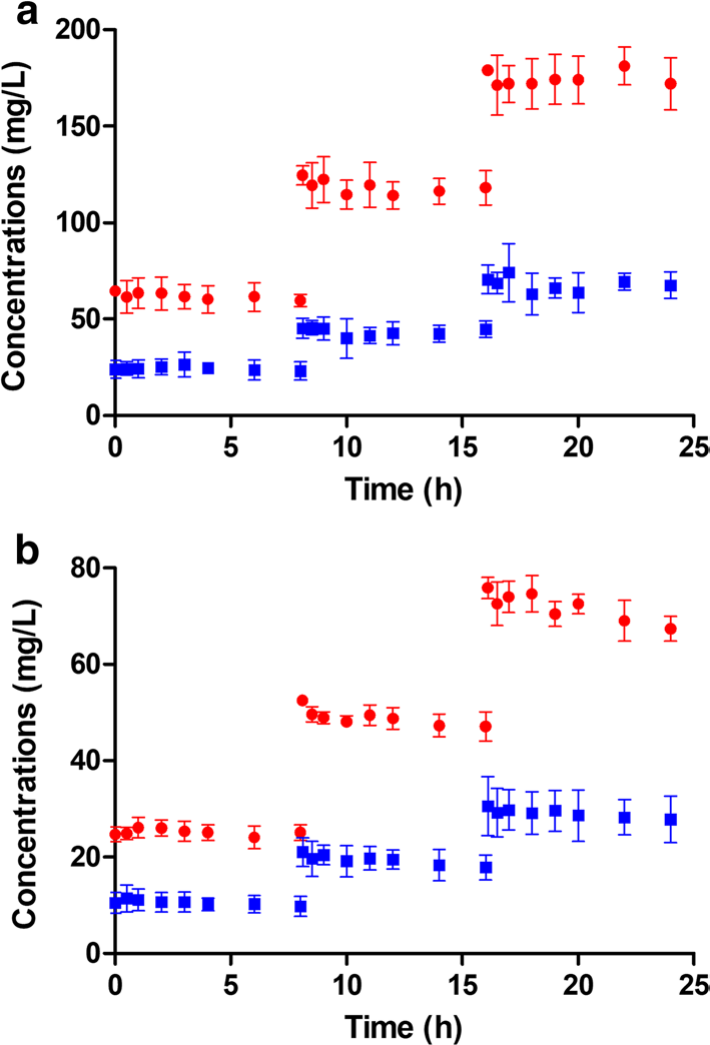
Concentrations observed during the ex vivo experiments. Mean ceftolozane (**a**) and tazobactam (**b**) concentrations observed in the low-concentration group (blue square) and the high-concentration group (red circle) with error bars representing standard error

For the two groups (low and high concentrations), ceftolozane and tazobactam concentrations increased respectively two- and three-fold after the second and the third administration, compared to the concentrations observed after the first administration (Fig. 1).

In the ECMO circuits, 8-h concentration variations ranged from − 5.73 to 1.26% and from − 12.95 to − 2.89% for ceftolozane and tazobactam, respectively. In the control tubes, 8-h concentration variations ranged from − 1.94 to 1.33% and from − 11.74 to − 5.14% for ceftolozane and tazobactam, respectively. No significant differences were observed between the loss in the ECMO circuits and in the control groups (Table 1).

**Table 1:** C/T concentration variations measured using the ex vivo model

Group	Ceftolozane		Tazobactam	
	8-h variation (%)	Dunnett test value	8-h variation (%)	Dunnett test value
Low concentration				
T0–T8	− 4.28	0.391	− 11.20	0.019
T8–T16	− 2.63	0.433	− 12.95	0.524
T16–T24	− 4.00	0.773	− 7.51	0.441
Control	− 1.94		− 11.74	
High concentration				
T0–T8	− 5.73	1.146	− 2.89	1.295
T8–T16	− 3.99	1.147	− 6.75	0.340
T16–T24	1.26	0.260	− 9.72	0.693
Control	1.33		− 5.14	

Ceftolozane/tazobactam concentration variations measured in low and high concentration groups during the T0–T8, T8–T16 and T16–T24 inter-dose intervals and in the control glass tubes

### Experiments on the in vivo model

**Fig. 2:**
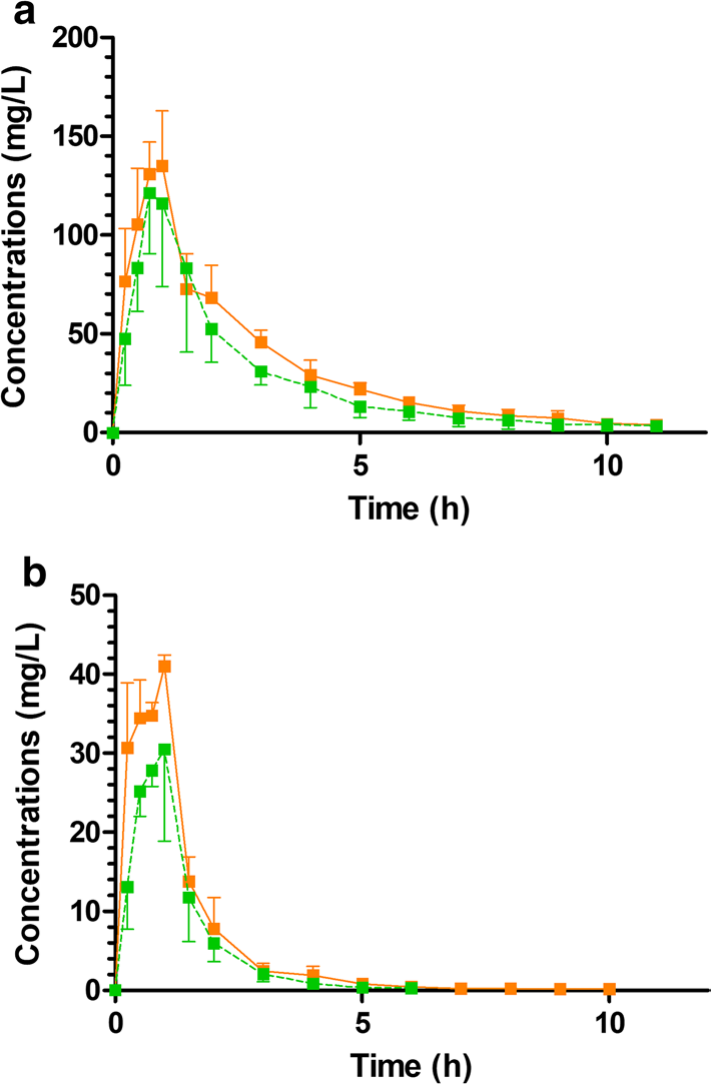
Plasma concentrations versus time measured during the in vivo study. Ceftolozane (**a**) and tazobactam (**b**) concentrations observed in the ECMO group (continuous orange line) and the control group (green dotted line). In order to ensure readability, only mean values are presented with half error bars representing standard error

A total of 96 serum samples from six pigs were collected and analyzed over 11 h. All 96 ceftolozane concentrations and only 71 tazobactam concentrations were measurable. Twenty-five tazobactam concentrations were below the LOQ (0.1 mg/l). Therefore, these concentrations were censored (concentrations in the 0–0.1 mg/l range without a precise value). Ceftolozane and tazobactam concentration profiles that were observed in ECMO and control groups are presented in Fig. 2.

### Pharmacokinetic analysis

Based on the non-compartmental approach, the mean of individual C/T parameters determined in the ECMO and the control group are presented in Table 2. For both molecules, means were not significantly different between the two groups (p > 0.05).

**Table 2:** Individual pharmacokinetic parameters determined for the in vivo model using a non-compartmental analysis

Parameter	Ceftolozane 2000 mg		Tazobactam 1000 mg	
	ECMO	Control	ECMO	Control
AUC (mg/h/l)	375.3 (14.6)	308.6 (34.9)	58.8 (10.7)	42.8 (20.5)
V_z_ (l)	20.85 (39.9)	31.31 (48.5)	34.93 (44.2)	29.35 (23.6)
V_ss_ (l)	15.33 (33.0)	19.09 (44.9)	13.88 (30.7)	16.99 (16.8)
Cl (l/h)	5.41 (15.7)	6.99 (32.0)	17.13 10.7)	24.98 (19.1)
T_1/2_ (h)	2.64 (32.0)	3.18 (40.1)	1.40 (42.9)	0.89 (40.5)

Mean (coefficient of variation) of parameters; *AUC* area under the curve; *V*_*z*_ volume of distribution during pseudo-equilibrium, *V*_*ss*_ volume of distribution at steady state, *Cl* elimination clearance, *T*_*1/2*_ elimination half-life

The best-fitting model to describe the PK profile of ceftolozane was a two-compartment model with a proportional error model (i.e. the higher the concentration, the greater the unexplained variability). The inter-individual variability of the central compartment volume (V_1_) was explained by sex (LRT = 11.02) while ECMO showed no effect on the inter-individual variability of any of the PK parameters. Estimations of the population parameters obtained with the final model are presented in Table 3.

**Table 3:** Estimated population parameters for ceftolozane using a modeling approach

Parameter	Model mean	RSE (%)
Fixed effects		
Cl (l/h)	6.15	9.83
V_1_ (l)	3.41	36.9
β_SEX/V1_	0.998	35.1
Q (l/h)	9.66	36.1
V_2_ (l)	9.92	15.2
BSV (%)		
Cl	23.5	31.4
V_1_	2.48	119
Q	68.7	36.8
V_2_	26.6	38.3
Residual variability (%)	13.9	8.77

*BSV* between-subject variability, expressed as the coefficient of variation of the associated non-log-transformed parameter, *RSE* relative standard error

For tazobactam, the best-fitting model was also a two-compartment model with a proportional error model. The only successful covariate was the ECMO that explains the inter-individual variability of the elimination clearance (Cl) (LRT = 6.14). Estimations of the population parameters obtained with the final model are presented in Table 4.

**Table 4:** Estimated population parameters for tazobactam using a modeling approach

Parameter	Model mean	RSE (%)
Fixed effects		
Cl (l/h)	24.2	7.86
β_ECMO/Cl_	− 0.304	35.2
V_1_ (l)	8.71	16.6
Q (l/h)	7.19	26.4
V_2_ (l)	6.37	14.1
BSV (%)		
Cl	11.3	44.9
V_1_	32.4	38.7
Q	31.9	44.5
V_2_	15.1	44.5
Residual variability (%)	19.9	10.8

*BSV* between-subject variability, expressed as the coefficient of variation of the associated non-log-transformed parameter, *RSE* relative standard error

More detailed results of the models developed are available in Additional file 1.

## Discussion

The use of antibiotics in critically ill patients is usually complex due to the extent of inter-individual variability of the pharmacokinetic parameters. One of the factors of variability is extracorporeal membrane oxygenation which induces considerable variability in antibiotic concentration for the same administered dose [13]. This point is particularly important as the pharmacokinetic–pharmacodynamic (PK–PD) criterion of beta-lactam efficacy depends on the percentage of the dosing interval that the free drug concentration remains above the minimal inhibitory concentration (% fT > MIC). For ceftolozane, the critical value of the PK–PD criterion, defined in in vitro and mouse models, ranges between 25 and 40% [14, 15]. To date and for ICU patients, the critical value is 100% fT > 4–6 MIC in order to ensure treatment efficacy regardless of the intra-individual PK variability [13]. Consequently, variations in the ceftolozane PK profile can significantly decrease the probability of target attainment.

The objective of our study was to document the influence of ECMO on the pharmacokinetics of C/T. First, we used an ex vivo model to characterize expected ceftolozane loss, according to: (1) the elapsed time of contact between the drug and the ECMO circuit and (2) the injected dose (low or high dose; one or repeated administrations). A complementary in vivo study was conducted using a porcine model with pharmacokinetic analysis (non-compartmental analysis and nonlinear mixed effect modeling).

The results obtained with the ex vivo (no significant loss compared to the control) as well as with the in vivo model suggest no consequence on treatment efficacy. Moreover, they pointed out two key elements that should be confirmed in clinical studies.

First, C/T adsorption was negligible, contrary to the results provided by Cies et al. [7]. With their ex vivo model, a ceftolozane loss of 40 to 60% was observed after 5 h and 90% after 24 h. In contrast, tazobactam concentrations were stable. Contradictory results have already been reported between ex vivo models for ceftriaxone [16, 17]. These contradictory results were explained by various hypotheses. In our study, the main hypothesis to explain those conflicting results is a difference in ECMO circuit materials. Cies et al. used one pediatric and one adult ECMO circuit with a peristaltic pump (Sorin Group, Liva Nova, London, United-Kingdom), a Quadrox-iD oxygenator (Maquet, Rastatt, Germany) and 1/4-in. Super-Tygon polyvinylchloride tubing [7]. A difference in coating seems to be the most likely explanation. The circuit used in our study has a phosphorylcholine coating while the Quadrox-iD membrane is heparin and albumin coated and SuperTygon tubing coating is not specified. This difference in coating could explain a difference in drug interactions with the surface leading to a difference in adsorption. Moreover, the chemical properties of ceftolozane support our results. Being hydrophilic with low protein binding (20%), ceftolozane should not adsorb on the ECMO circuit.

The second point of interest is the influence of ECMO on the renal clearance of C/T. In our study, ECMO induced a decrease in tazobactam clearance (17.9 vs. 24.2 l/h) based on the modeling approach, and of ceftolozane clearance (5.41 vs. 6.99 l/h) according to a non-compartmental analysis. Since both molecules are eliminated mostly by the kidneys (> 95% and > 80% for ceftolozane and tazobactam, respectively), a decrease in total clearance suggests a decrease in renal clearance. Three mechanisms control renal excretion: (i) glomerular filtration (GFR), (ii) secretion involving transporters located on the proximal convoluted tubule, and (iii) reabsorption, by passive diffusion in the distal convoluted tubule. Because venoarterial ECMO induces continuous renal blood flow, while it is physiologically pulsatile [18], it is likely that ECMO modifies renal blood flow and therefore, the GFR [19]. However, the impact on secretion and reabsorption is less evident. In fact, secretion mechanisms are unlikely to be modified in the absence of co-prescription or endogenous substance accumulation, while reabsorption depends on urine pH. This physiological information was lacking in our study. Consequently, if a decrease in ECMO-related clearance is suspected in patients, a slight over-exposure of ceftolozane and tazobactam can be expected, without any severe adverse effects.

For the in vivo experiments, a porcine model was chosen because of anatomical similarities (vascular volume, blood flow, etc.), which provided pigs compatible with the human adult ECMO device, and with physiological similarities that make them an interesting human pharmacokinetic model [20, 21]. A two-compartment model was selected for both ceftolozane and tazobactam as was the case in studies performed in humans [22, 23]. Clearance values reported in those studies are similar to our results (4.84 and 6.3 vs. 6.15 l/h; and 16.4 and 24.5 vs. 20.9 l/h for ceftolozane and tazobactam, respectively), which confirms the relevance of the pig model to describe drug elimination in humans.

While to our knowledge, this is the first study to investigate the pharmacokinetics of C/T with ECMO using both an ex vivo and an in vivo model, this study has several limits. Firstly, this was a pilot study with a small number of animals (n = 6) and a low representativeness of what could happen in a larger population. Secondly, we did not induce sepsis and/or pneumonia in the pigs, which is unlike critically ill patients suffering from sepsis or severe ARDS and who require ECMO. In fact, this process is much too complex for this type of pilot study. Moreover, despite an apparent simplicity, this pilot study was highly time-consuming and required a well-trained multidisciplinary team. Consequently, our results do not completely reflect the pharmacokinetic changes in this specific population.

## Conclusions

Using an ex vivo and an in vivo porcine model, our study provides preliminary evidence that normal ceftolozane and tazobactam dosing in ICU patients should be effective in patients undergoing ECMO. Nevertheless, clinical data are required to confirm and validate these findings in order to implement dosing guidelines.

## Supplementary information

**Additional file 1.** More precisions about modeling methodology and results.

## Data Availability

The datasets analyzed during the current study are available from the corresponding author on reasonable request.

## Abbreviations

C/T: Ceftolozane/tazobactam
ECMO: Extracorporeal membrane oxygenation
GFR: Glomerular filtration rate
ICU: Intensive care unit
LRT: Likelihood ratio test
PK: Pharmacokinetics
PK–PD: Pharmacokinetics–pharmacodynamics
