# Supplementary material for: Influence of extracorporeal membrane oxygenation on the pharmacokinetics of ceftolozane/tazobactam: an ex vivo and in vivo study
Source: J Transl Med. 2020 May 27;18:213. doi: 10.1186/s12967-020-02381-1 (PMC7251674; doi:10.1186/s12967-020-02381-1)

Influence of Extracorporeal Membrane Oxygenation on the Pharmacokinetics of Ceftolozane/Tazobactam

Camille Mané, Clément Delmas, Jean Porterie, Géraldine Jourdan, Patrick Verwaerde, Bertrand Marcheix, Didier Concordet, Bernard Georges, Stéphanie Ruiz, Peggy Gandia

Online Data Supplement

Methods of pharmacokinetic analysis

Non-compartmental analysis

Pharmacokinetic interpretation of the *in vivo* ceftolozane/tazobactam (C/T) concentrations versus time was performed using PK Solver software (E1). The elimination rate was estimated by linear regression using the last five points of the kinetic profile.

Nonlinear mixed effect modeling

The modeling approach applied to the *in vivo* C/T concentrations was performed with MONOLIX software 2018 R2 (Lixoft, Antony, France). Means of individual pharmacokinetic parameters determined by non-compartmental analysis were used as initial values to estimate the population pharmacokinetic parameters. Concentrations below the lower limit of quantification (LOQ) were used with censoring for modeling.

One and two-compartment structural models associated with first-order elimination were tested using an exponential inter-individual variability model (Equation 1).

$$\theta_i = \theta_{pop} * e^{\eta_i} \qquad (1)$$

Where: θ_i is the estimated individual pharmacokinetic parameter for the ith individual, θ_{pop} is the median value of the pharmacokinetic parameter of the population, and η_i is the inter-individual random effect for the ith individual assumed to be normally distributed with a mean of 0 and a variance of ω^2.

An initial combined proportional-additive residual error model was tested. (Equation 2)

$$Y_{O,ij} = Y_{P,ij} \,.\, \left(1 + \varepsilon_{prop,ij}\right) + \varepsilon_{add,ij} \qquad (2)$$

Where:

$Y_{O,ij}$ and $Y_{P,ij}$ are the observed and predicted jth drug concentrations for the ith individual respectively.

$\varepsilon_{prop,ij}$ and $\varepsilon_{add,ij}$ are the proportional and additional error respectively, with a mean of 0 and a variance of σ^2_{prop} and σ^2_{add} respectively. In case of negligible $\varepsilon_{add,ij}$ estimated value, a residual error model was adapted in a proportional error model.

The influence of ECMO and gender were tested as dichotomic covariates on θ_i. (Equation 3)

$$\theta_i = \theta_{pop} \,.\, e^{\beta_{dic} \cdot DIC_i} \qquad (3)$$

Where: θ_i is the estimated individual pharmacokinetic parameter for the ith individual, θ_{pop} is the median value of the pharmacokinetic parameter of the population, DIC_i is the dichotomic covariate (0 or 1) for the ith individual and β_{dic} quantifies the influence of the associated covariate.

The evaluation of the null model was based on the usual criteria: improvement of the likelihood (-2LL), precision of the PK parameter estimation (relative standard error, RSE) and diagnostic plot evaluation (observed *vs.* population predicted concentrations scatter plot,

observed *vs.* individual predicted concentrations scatter plot and residual plots (individual weighted residuals (IWRES) *vs.* time and predicted concentrations))

The significant influence of a covariate to explain inter-individual variability was determined applying the Wald test, the likelihood ratio test (LRT) and the improvement of the Akaike Information Criterion (AIC) and the Bayesian Information Criterion (BIC). A decrease of more than 3.84 in the criteria (p-value = 0.05, χ^2 distribution, 1 degree of freedom) was considered as significant. Statistical significance of studied covariates was assessed by stepwise inclusion only due to the limited number of tested covariates.

Suitability of the final model was evaluated by visual predictive check (VPC).

Results of nonlinear mixed effect modeling

Ceftolozane

A two-compartment model described ceftolozane kinetic profiles more adequately than a one-compartment model (Figure E1: top of the figure). The best-fitting error model was a proportional one (Figure E1: bottom of the figure) due to the negligible estimated additive error in the combined error model ($2.22 .10^{-16}$). The choice of a two-compartment structural model with a proportional error model compared to a one-compartment model with a combined error model was confirmed by the likelihood (111.20 *vs.* 249.75), the AIC (129.20 *vs.* 261.75) and the BIC (127.32 *vs.* 260.51) values.

Only gender was a significant covariate (coded with 1 for male and 0 for female) to explain the inter-individual variability of the central compartment volume V_1 (2.48% *vs.* 59%) with a significant Wald test (p = 0.004), a LRT of 11.02, a decrease in the AIC (120.18 *vs.* 129.2) and the BIC (118.1 *vs.* 127.32) values. On the other hand, ECMO was not found to be

a significant covariate for any parameter. The VPCs were acceptable for the cohort size (Figure E2).

Tazobactam

As was the case for ceftolozane, a two-compartment model described tazobactam kinetic profiles more adequately than a one-compartment model (Figure E3: top of the figure). The best-fitting error model was also a proportional one (Figure E3: bottom of the figure). The choice of a two-compartment structural model with a proportional error model compared with a one-compartment model with a combined error model was confirmed by the likelihood (- 204.88 *vs.* - 63.44), the AIC (- 186.88 *vs.* - 53.44) and the BIC (- 188.76 *vs.* - 54.48) values.

ECMO was a significant covariate (1 for presence and 0 for absence) to explain the inter-individual variability of clearance (11.3% *vs.* 19%) with a significant Wald test (p = 0.004), a LRT of 6.14, a decrease in the AIC (-191.02 *vs.* -186.88) and the BIC (-193.10 vs -188.76) values. The VPCs based on the ECMO status were acceptable for the cohort size (Figure E4).

References

E1. Zhang Y, Huo M, Zhou J, Xie S. PKSolver: An add-in program for pharmacokinetic and pharmacodynamic data analysis in Microsoft Excel. *Comput Methods Programs Biomed* 2010;99:306–314.

Figures

Figure E1: Goodness-of-fit plots for the final ceftolozane model. Top of the figure: observed concentrations versus population-predicted (A) and individual-predicted (B) concentrations. Bottom of the figure: individual weighted residuals versus time (C) and versus individual-predicted concentrations (D)

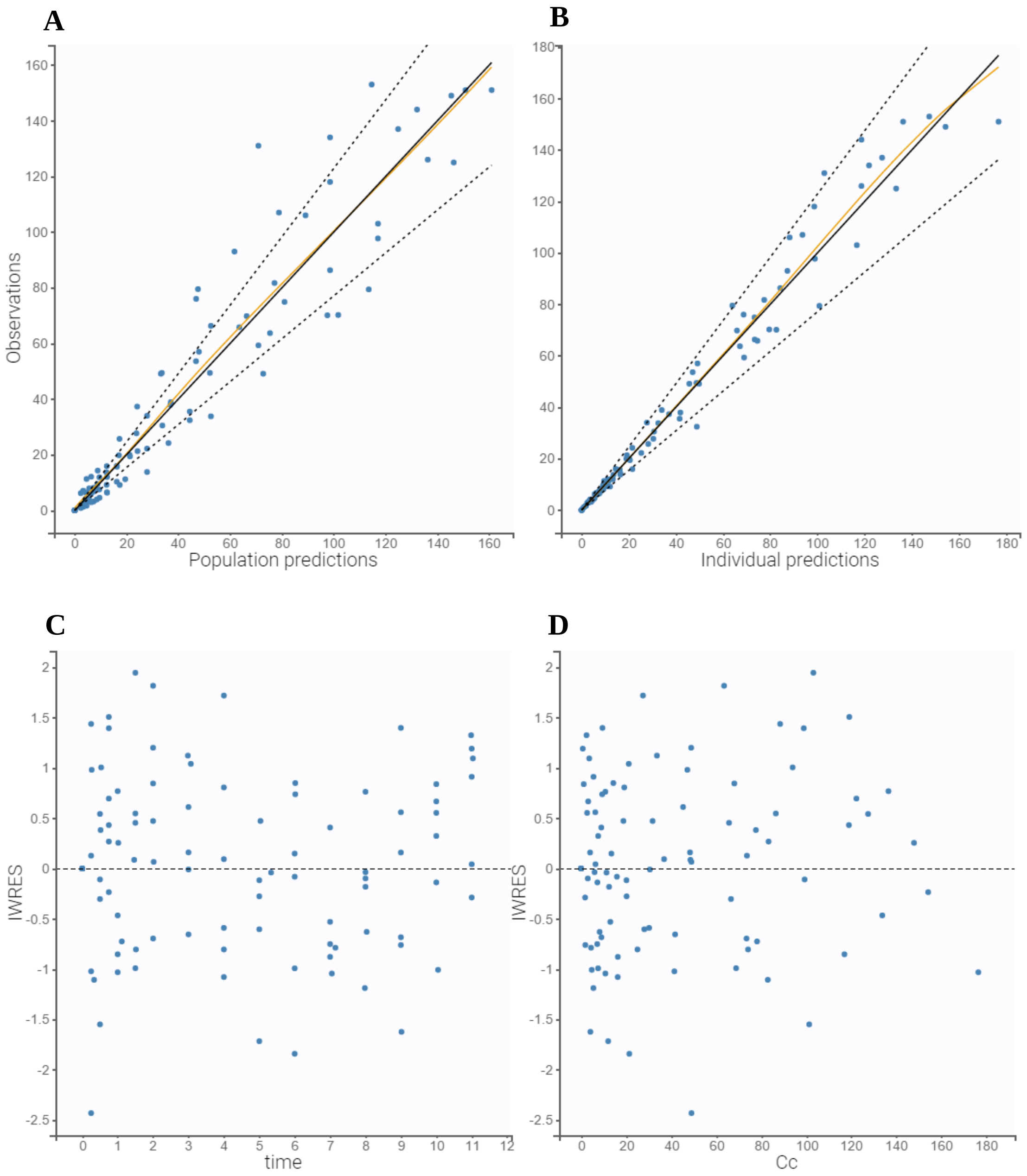

Figure E2: Visual Predictive Check of the final ceftolozane model. The observed data (black circle) were plotted with the median (black line), 10th and 90th percentiles of the predictions (grey lines). The 90% confidence intervals of the 10th and 90th percentiles are represented by the shaded grey area.

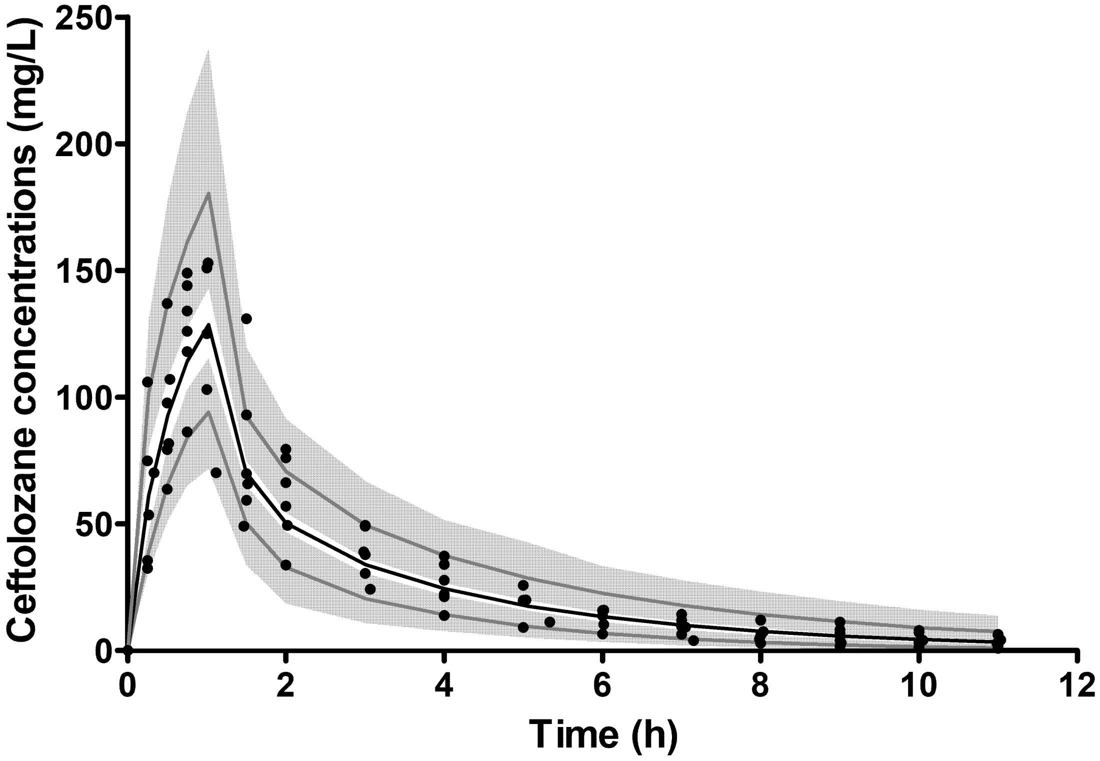

Figure E3: Goodness-of-fit plots for final tazobactam model. Top of the figure: observed concentrations versus population-predicted (A) and individual-predicted (B) concentrations. Bottom of the figure: individual weighted residuals versus time (C) and versus individual-predicted concentrations (D). The blue dots represent the measured concentrations while the red dots are censored values (i.e. values between 0 and the lower limit of quantification).

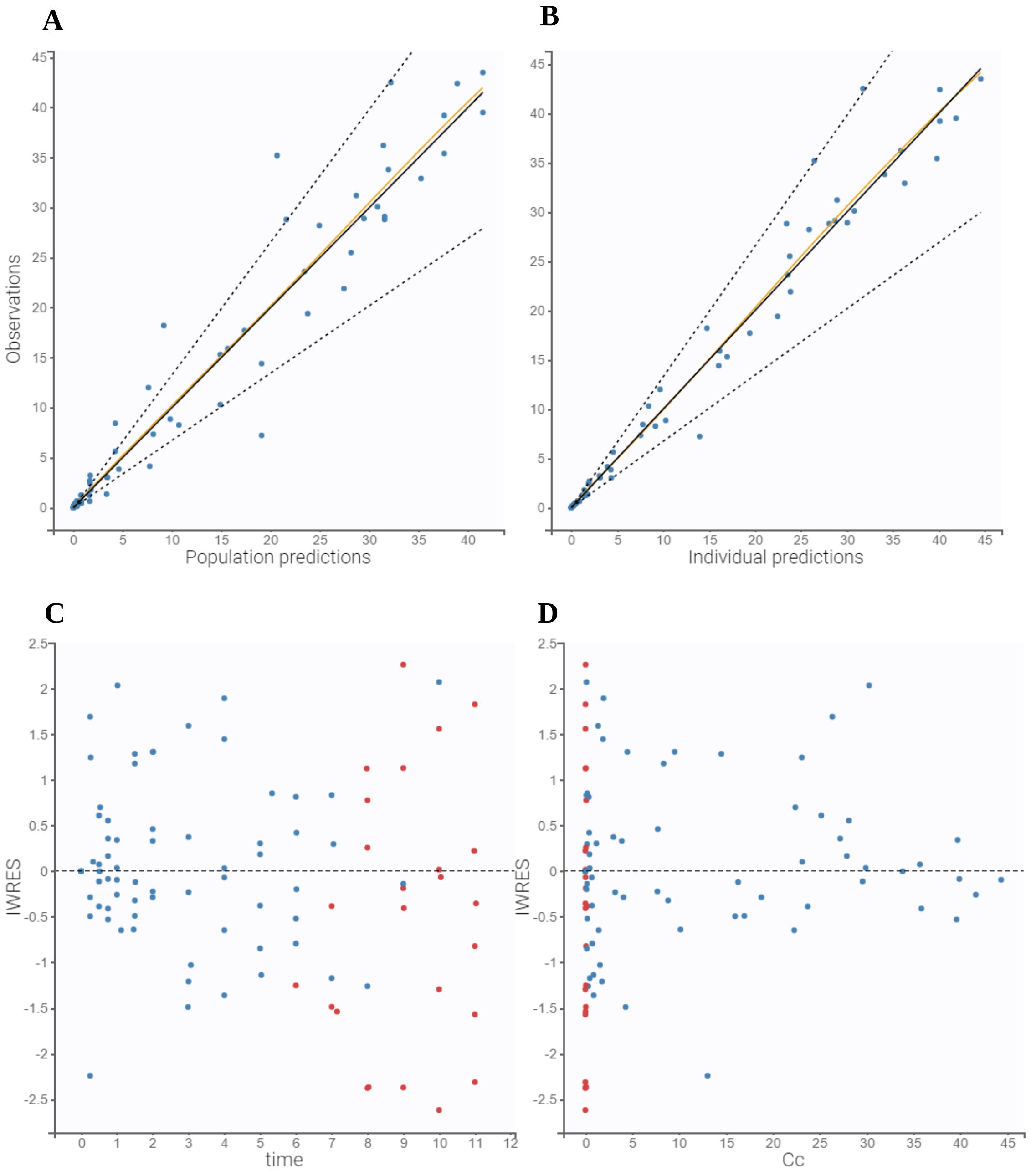

Figure E2: Visual Predictive Check of the final tazobactam model. The observed data (black circle) were plotted with the median (black line), 10th and 90th percentiles of the predictions (grey lines). The 90% confidence intervals of the 10th and 90th percentiles are represented by the shaded grey area.

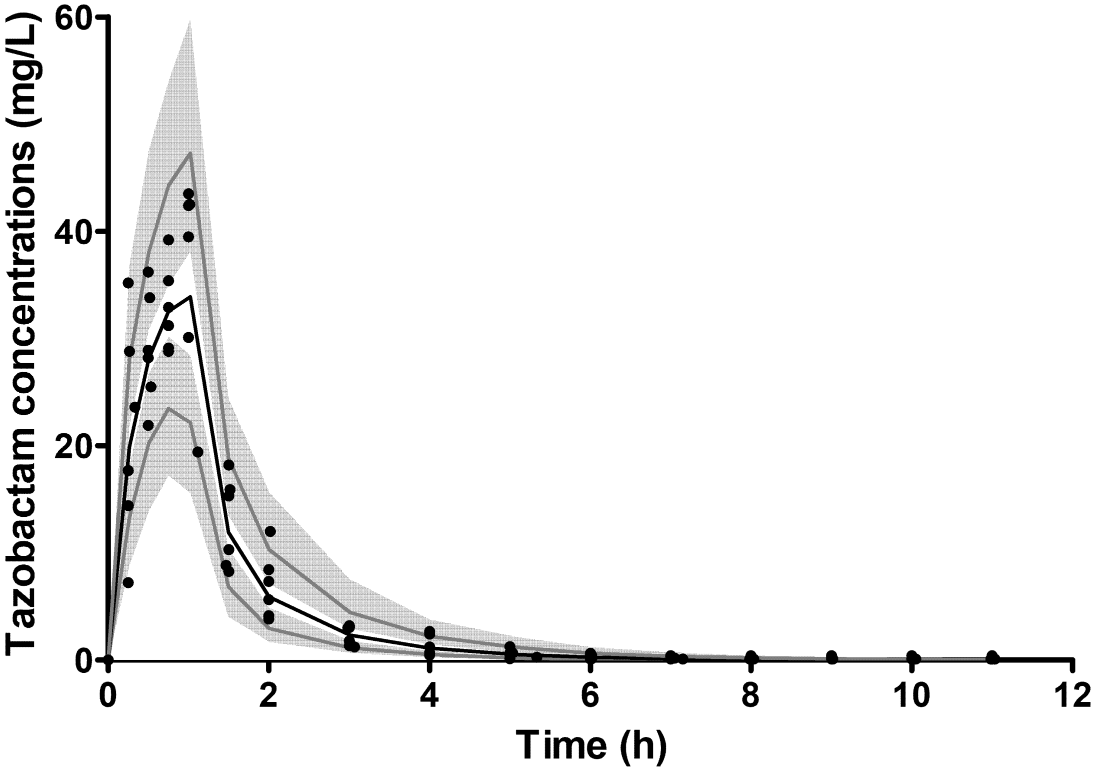

Supplement: Supplementary file 1 — Additional file 1. More precisions about modeling methodology and results. [file 12967_2020_2381_MOESM1_ESM.pdf]
